# Transvesical Retzius-Sparing Versus Standard Robot-Assisted Radical Prostatectomy: A Retrospective Propensity Score-Adjusted Analysis

**DOI:** 10.3389/fonc.2021.687010

**Published:** 2021-05-17

**Authors:** Wen Deng, Hao Jiang, Xiaoqiang Liu, Luyao Chen, Weipeng Liu, Cheng Zhang, Xiaochen Zhou, Bin Fu, Gongxian Wang

**Affiliations:** ^1^ Department of Urology, The First Affiliated Hospital of Nanchang University, Nanchang City, China; ^2^ Jiangxi Institute of Urology, Nanchang City, China

**Keywords:** prostate cancer, robot-assisted radical prostatectomy, Retzius-sparing, transvesical approach, standard approach

## Abstract

**Objectives:**

To estimate the safety and efficiency of transvesical Retzius-sparing robot-assisted radical prostatectomy (T-RARP) compared with standard robot-assisted radical prostatectomy (S-RARP) for localized prostate cancer (PCa).

**Materials and Methods:**

174 patients bearing localized PCa and undergoing T-RARP or S-RARP between October 2017 and January 2020 were retrospectively enrolled in our analysis. All potential baseline confounders were strictly restrained with propensity-score matching (PM) method (1: 1). Within the matched setting, the perioperative and functional outcomes were compared between the T-RARP and S-RARP groups, while the oncological results and functional recovery of the two arms were presented with Kaplan-Meier curves.

**Results:**

Finally, 114 and 60 eligible patients harbouring localized PCa were identified in the S-RARP and T-RARP group, respectively. No significant differences between the two groups were found in all baseline characteristics after PM. Within the matched cohort, no case was converted to open surgery in either group. The T-RARP group was significantly related to a higher mean operative time (*p* = 0.001) and shorter median hospital stay length (*p* < 0.001). There were not significant differences in the median estimated blood loss and specimen Gleason score between the two arms. The proportions of transfusion, pT3a disease, postoperative complication, and positive surgical margin in the T-RARP group were also comparable to that in the S-RARP group. The mean prostate-specific antigen and median erectile functional scores did not differ significantly between the two groups at postoperative 3 months and last follow-up. T-RARP vs. S-RARP had significantly improved urinary continence (UC) rates at the removal of catheter (*p* < 0.001) and postoperative 3 months (*p* < 0.001), but the significant difference between the two groups in UC recovery disappeared at last follow-up (*p* = 0.119). No significant difference in biochemical recurrence-free survival was observed following the two surgeries (*p* = 0.727).

**Conclusions:**

T-RARP by experienced hands was feasible for selected patients with clinically localized PCa, yielding significantly improved early return to UC and similar erectile functional preservation without compromising oncological control when compared with the standard approach.

## Introduction

The proportion of patients afflicted with localized prostate cancer (PCa) was sharply improved along with the wide dissemination of serum prostate-specific antigen (PSA)-based screening (1). Radical prostatectomy (RP), an option with improved overall and cancer-specific survival benefits when compared with watchful waiting for localized PCa (2), aims to eradicate localized PCa whilst, whenever possible, preserve urinary continence (UC) and erectile function (EF), namely, a trifecta outcome. Given the complexity of the anatomy of prostate and its surrounding structures and the executive facilitation of robotic surgery, robot-assisted RP (RARP) has been adopted in a diffuse manner since it was firstly conducted in 2001 and currently represented the most common surgical procedure for prostatectomy (3).

Over the past 2 decades, various surgical approaches have been implemented with the aim of optimizing the functional preservations following RARP, such as the standard/anterior, posterior, lateral, and transperineal method. Among them, the standard/anterior and posterior approaches are the two main choices to perform RARP. The posterior RARP described by Bocciardi et al. (4), known as the “Retzius-sparing” method, approaches to prostate gland through Douglas’ pouch and avoids any disturbance to the related anatomical structures in Retzius’ space, thus resulting in a faster UC recovery (5). However, the dissemination of posterior RARP has been hindered due to its steep learning curve and the scarcity of valid evidences to confirm the reproducibility of short-term outcomes, the exhibition of improved long-term outcomes and the identification of oncologic efficacy (6–8). The transvesical approach to RARP (T-RARP) was merely applied on two cadavers with the da Vinci-S robotic system before, exhibiting the technical feasibility in human body (9). Similar to the posterior RARP, T-RARP obviated any disruptions to the Retzius space, which is of great importance to optimum postoperative continence. Our team firstly performed T-RARP, another approach to Retzius-sparing RARP, on well-selected patients with localized PCa and detailedly reported its whole procedure on a multi-port basis (10).

Given the advantage of T-RARP in preserving the Retzius’ space over S-RARP, we assumed that T-RARP could also obtain better UC recovery than S-RARP when surgically treating localized PCa. Here, we conducted the first study concentrating on comparing the impacts of T-RARP and S-RARP on perioperative results, preservation of UC and EF, and cancer control with propensity-score matching (PM) method.

## Materials and Methods

### Data Source and Ethics Statement

Our prospectively maintained database was retrospectively scrutinized to acquire details regarding the baseline demographic, clinical and pathological information after obtaining the approval of the institutional review board and ethics committee of the First Affiliated Hospital of Nanchang University.

### Patients Selection

All patients with primary localized PCa between October 2017 and January 2020 were reviewed and included in the final comparison only when they underwent T-RARP or S-RARP, while those with a history of abdominal surgery were excluded from our analysis. All cases were routinely checked with preoperative prostate magnetic resonance imaging, bone scintigraphy, and abdominal computed tomography scan.

The patients in the S-RARP arm were usually assigned at the surgeons’ discretion on the basis of tumor and patient characteristics. As described in our previously published study (10), only patients with low- or immediate-risk PCa and prostate volume ≤ 80 mL were considered suitable for T-RARP at current stage, and these candidates were discretionarily enrolled in the T-RARP arm after meticulous descriptions of why and how to perform T-RARP, the distinctions between different approaches to RARP and alternative choices of cancer management, and following provided the written informed consent including all information mentioned above. All surgeries were done after the acquisition of informed consent.

### Technical Considerations

The standard approach was done in line with the techniques established by Menon (5, 11), and all patients in the S-RARP group underwent RARP with posterior reconstruction. While the transvesical approach was performed since January 2018 in compliance with the surgical procedures detailedly presented in our previously published study (10). Several technically pivotal points should be highlighted to better understand this novel surgical technique (T-RARP) we firstly applied on patients with PCa. It’s helpful to expose the operative field with percutaneous suspension stitches expending the cystotomy from both sides. A third-arm Prograsp could be very helpful to retract the isolated vas deferens and seminal vesicles upwards when dissecting the posterior aspect of the prostate. The initial dissection during T-RARP starts posteriorly, which is similar to the procedure of the posterior Retzius-sparing RARP. The technique regarding urethrovesical anastomosis is almost identical to the standard/anterior approach with which we are familiar. The “dead space” around the anastomotic location was routinely filled with fibrin-based haemostatics.

All procedures in both groups were completed by two highly experienced surgeons (Wang GX and Fu B) who had received standardized training and conducted more than 300 RARP *via* the anterior/standard method by the time that they firstly applied the transvesical approach to RARP on patient with low-risk PCa. Only when the preoperative estimated risk of finding nodal metastases exceeded 5% were extended pelvic lymph nodes (ePLND) dissected, while other cases were surgically managed without nodal dissections due to the low risk of missing involved nodes. The nerve-sparing technique was routinely performed in a similar manner for low- and immediate-risk patients in both groups, while for those with high-risk PCa it was preoperatively proposed according to clinical features and intraoperatively modified based on evidence of bundle invasion.

### Variable Definition and Endpoints

Patients satisfying the inclusion criteria were divided into the T-RARP and S-RARP groups according to the surgical type. The following preoperative variables were retrieved for each patient: age, body mass index (BMI), diabetes mellitus, hypertension, American Society of Anesthesiologists (ASA) score, preoperative serum total PSA, prostate volume calculated by virtue of transrectal ultrasound, preoperative EF evaluated according to the International Index of Erectile Function (IIEF)-5 score (12), clinical TNM stage, and biopsy Gleason score. Perioperative variables consisted of operative time (OT), estimated blood loss (EBL), conversion (to open surgery), transfusion, postoperative complications graded according to the Clavien-Dindo classification (13), urethral catheterization length, and postoperative hospital stay. Pathologic outcomes included pathological staging, specimen Gleason score, and positive surgical margin (PSM). The follow-up arrangements were postoperatively regular for each patient. PSA tests were done routinely every 3 months after surgery to monitor biochemical recurrence (BCR), which was diagnosed on the first occasion after prostatectomy that two consecutive rising serum PSA was 0.2 ng/mL or greater. Data with regard to UC, which was defined as the avoidance of any pads or preventively requiring one dry pad over the 24-hour period, was also compared at removal of catheter and postoperative 3 months and 12 months. EF was considered as recovered when postoperative IIEF score was 22 or greater.

### Statistical Analysis

A PM analysis was performed to eliminate the impact of significant differences in preoperative clinical characteristics between the T-RARP and S-RARP groups. All preoperative features were taken into account to estimate the propensity score *via* applying non-parsimonious and multivariate logistic regression. Finally, 60 patients in the S-RARP group were successfully matched to patients treated with T-RARP in a 1:1 ratio in accordance to the nearest neighbor matching method within the matching strategy. The covariate differences were compared before and after matching to delineate the improved balance between the procedure arms after PM.

All normally distributed continuous variables were presented as mean and standard deviation (SD) and analyzed with the independent t-test, while other non-normally distributed ones were described as median and interquartile range (IQR) and compared employing the Wilcoxon-rank sum test. Categorical features were calculated relying on the Pearson chi-squared or Fishers’ exact test. The Kaplan-Meier method was employed before and after PM to estimate the biochemical recurrence (BCR) - free survival using log-rank test. The STATA version 12.0 (STATA corp., College Station, TX) was utilized to perform all statistical analyses. All statistical tests were performed two-sided at the 5% significance level.

## Results

**Table 1** has detailedly depicted the preoperative demographic and tumor characteristics before and after matching. In all, 114 and 60 patients with primary localized PCa fulfilling the inclusion criteria were enrolled in the S-RARP and T-RARP arms, respectively, within the period when being reviewed. All statistically significant differences in preoperative parameters disappeared within the well-balanced matched cohorts after PM (**Table 1**).

**Table 1:** Preoperative characteristics by surgery type before and after propensity score matching.

Variable	Before propensity score matching			After propensity score matching		
	S-RARP (*n* = 114)	T-RARP (*n* = 60)	*p* value	S-RARP (*n* = 60)	T-RARP (*n* = 60)	*p* value
Age, years, mean (SD)	67.3 (7.5)	63.3 (7.3)	0.001	64.9 (8.0)	63.3 (7.3)	0.271
BMI, kg/m2, mean (SD)	22.5 (3.8)	23.2 (3.7)	0.257	23.2 (3.4)	23.2 (3.7)	0.907
Diabetes mellitus (yes), n (%)	17 (14.9%)	9 (15.0%)	0.988	11 (18.3%)	9 (15.0%)	0.624
Hypertension (yes), n (%)	32 (28.1%)	20 (33.3%)	0.471	18 (30.0%)	20 (33.3%)	0.695
ASA score (≥ 3), n (%)	10 (8.8%)	4 (6.7%)	0.774	5 (8.3%)	4 (6.7%)	1
Preoperative total PSA, ng/mL, mean (SD)	26.0 (12.9)	19.5 (6.1)	0.001	20.1 (6.8)	19.5 (6.1)	0.582
Prostate volume, mL, mean (SD)	42.9 (13.1)	36.8 (9.5)	0.001	39.5 (9.8)	36.8 (9.5)	0.472
Preoperative IIEF-5 score, median (IQR)	16 (13, 19)	17 (14, 20)	0.028	18 (14.3, 19)	17 (14, 20)	0.641
cTNM stage, n (%)			0.001			0.564
T1c	49 (43.0%)	22 (36.7%)		19 (31.7%)	22 (36.7%)	
T2a-b	48 (42.1%)	38 (63.3%)		41 (68.3%)	38 (63.3%)	
T2c	17 (14.9%)	0 (0%)		0 (0%)	0 (0%)	
Biopsy Gleason score, median (IQR)	7 (6, 8)	6 (5, 7)	0.001	6 (5, 7)	6 (5, 7)	0.635

SD, standard deviation; BMI, body mass index; ASA, American Society of Anesthesiologists; IIEF, International Index of Erectile Function; IQR, inter-quartile range.

Perioperative and pathological outcomes were listed in **Table 2**. Within the matched cohorts, all surgeries were successfully conducted without any open conversion in either arm. The mean OT in the T-RARP group was significantly longer than that in the S-RARP group (134.2 vs 110.0 min, *p* = 0.001), while the statistical difference in the median EBL was not significant between the two groups (110.7 vs. 97.8 ml, *p* = 0.237). 8 (13.3%) and 5 (8.3%) cases received extend PLND in the S-RARP and T-RARP groups, respectively (*p* = 0.378), and none of them in either group was detected with involving lymph nodes. The percentages of transfusion, ≤ Grade II and > Grade II complications did not differ significantly between the two groups (*p* = 1.000, *p* = 0.543, and *p* = 0.496, respectively). Urinary retention after catheter removal was noted in one case in either group and successfully managed by catheter insertion, and none of them complained of secondary damage to vesicourethral anastomosis integrity due to the catheter insertion. The statistical significances for all pathological outcomes concerning pathologic stage, specimen Gleason score and PSM were confirmed with a two-sided *p* > 0.05 after the PM (*p* = 0.102, *p* = 0.079, and *p* = 0.591, respectively). Only 4 and 1 patients harbored pathologic T3a diseases in the S-RARP and T-RARP groups, respectively. 2 and 1 PSMs were detected in these pT3a cases in the S-RARP and T-RARP groups, respectively. Thanks to the avoidance of pelvic drainage placement and the faster removal of catheter after T-RARP in a routine manner, patients in the T-RARP arm had a significantly shorter median hospital stay length than that in the S-RARP arm (8 vs. 14 days, *p* = 0.001).

**Table 2:** Perioperative outcomes for S-RARP and T-RARP after propensity score matching.

Variable	S-RARP (*n* = 60)	T-RARP (*n* = 60)	*p* value
Operative time, min, mean (SD)	110.0 (29.4)	134.2 (27.0)	0.001
Estimated blood loss, mL, mean (SD)	97.8 (50.7)	110.7 (66.4)	0.237
ePLND, n (%)	8 (13.3%)	5 (8.3%)	0.378
Open conversion, n (%)	0 (0%)	0 (0%)	–
Transfusion, n (%)	1 (1.7%)	0 (0%)	1
Postoperative pathology			
Pathological T stage, n (%)			0.364
pT2	56 (93.3%)	59 (98.3%)	
pT3a	4 (6.7%)	1 (1.7%)	
Specimen Gleason score, median (IQR)	7 (5, 7)	6 (5, 7)	0.079
Positive surgical margin, n (%)	7 (11.7%)	9 (15.0%)	0.591
Postoperative complications, n (%)	9 (15.0%)	5 (8.3%)	0.255
≤ Grade II complications	7 (11.7%)	5 (8.3%)	0.543
> Grade II complications	2 (3.3%)	0 (0%)	0.496
Urethral catheterization, days	14	7	–
Hospital stay, days, median (IQR)	14 (14, 15)	8 (7, 8)	< 0.001

ePLND, extended pelvic lymph nodes dissection; SD, standard deviation; IQR, inter-quartile range.

**Figure 1:**
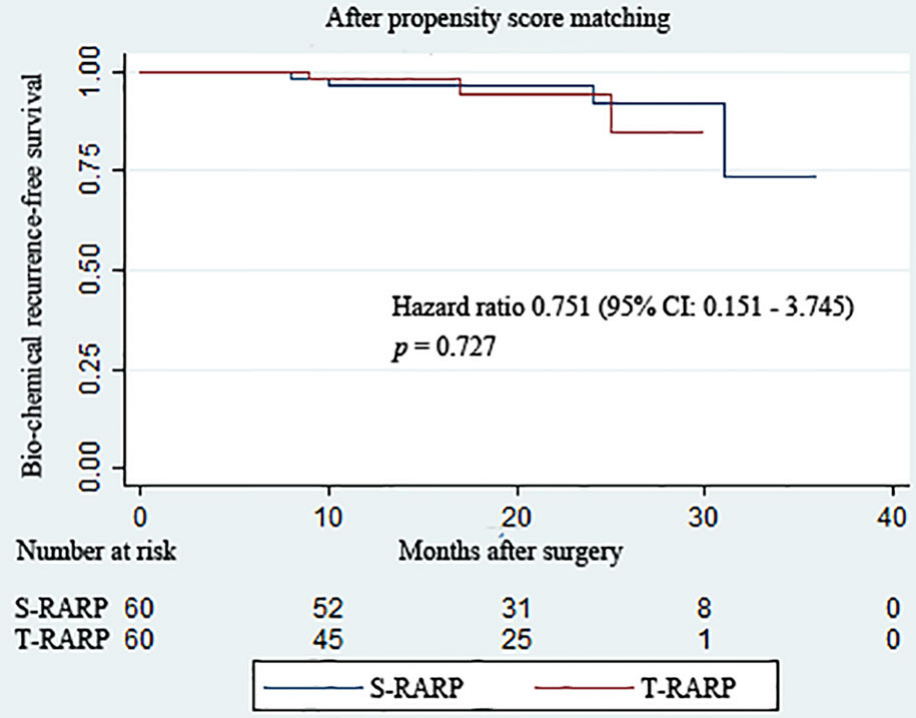
Kaplan–Meier curves showing biochemical recurrence−free survival for patients undergoing the transvesical and standard approaches to robot-assisted radical prostatectomy during the follow-up intervals.

All patients in both groups were regularly followed up for at least 12 months postoperatively. The median follow-up periods of the S-RARP and T-RARP groups after PM were 20.0 and 14.0 months, respectively. Within the matched cohort, the results with regard to the mean total serum PSA at postoperative 1 week, 3 months, and last follow-up indicated comparable outcomes between the two groups (**Table 3**). The incidences of BCR have occurred to four and three patients in the S-RARP and T-RARP groups, respectively, during the follow-up intervals. Among those with pT3a diseases, the occurrence of BCR has happened to one patient in either group of our analysis, respectively. The statistical difference in BCR remained insignificant after PM between the two groups (*p* = 0.727) (**Figure 1**).

**Table 3:** Postoperative outcomes for S-RARP and T-RARP after propensity score matching.

Variable	S-RARP (*n* = 60)	T-RARP (*n* = 60)	*p* value
Oncology: postoperative total PSA, ng/mL			
Postoperative 1 week, mean (SD)	1.614 (1.363)	2.072 (1.938)	0.178
Postoperative 3 months, mean (SD)	0.045 (0.026)	0.034 (0.130)	0.542
Last follow-up, mean (SD)	0.031 (0.015)	0.027 (0.021)	0.642
Urinary continence			
Continent on removal of catheter, n (%)	18 (30.0%)	54 (90.0%)	< 0.001
Continent at postoperative 3 months, n (%)	35 (58.3%)	60 (100%)	< 0.001
Continent at postoperative 12 months, n (%)	56 (93.3%)	60 (100%)	0.119
Continent at last follow-up, n (%)	56 (93.3%)	60 (100%)	0.119
Erectile function			
IIEF-5 score at postoperative 3 months, median (IQR)	14 (10, 18)	13 (9, 17)	0.322
IIEF-5 score at last follow-up, median (IQR)	13 (10, 17)	13 (9, 16)	0.471

PSA, prostate specific antigen; SD, standard deviation; IQR, inter-quartile range; IIEF, International Index of Erectile Function.

**Figure 2:**
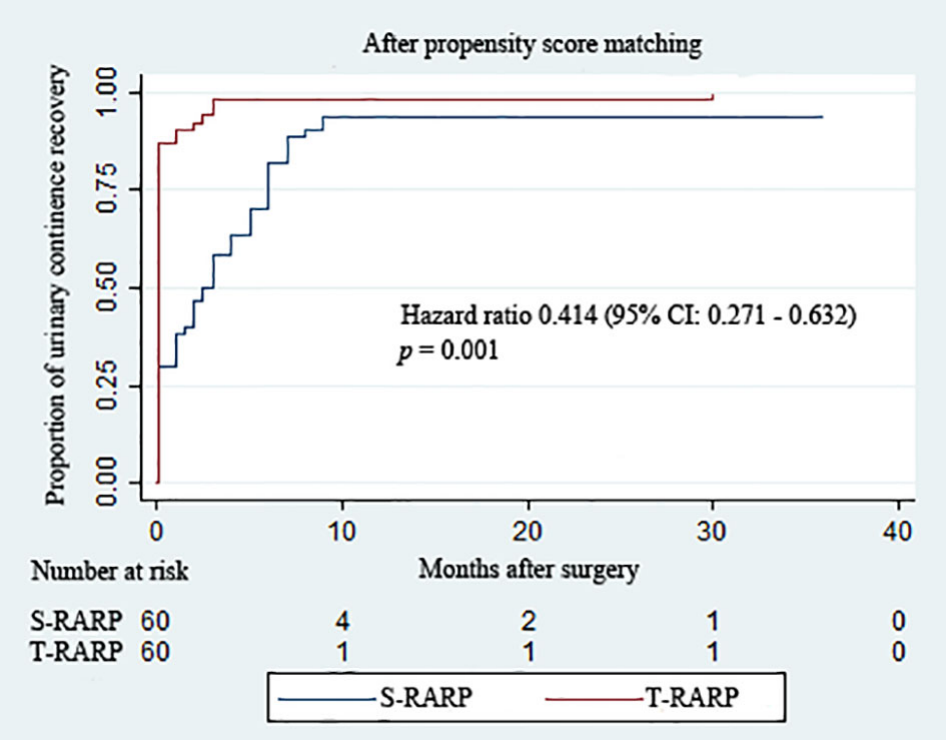
Kaplan–Meier curves showing the proportion of urinary continence (UC) in patients undergoing the transvesical and standard approaches to robot-assisted radical prostatectomy during the follow-up intervals. UC was defined as requiring no pad or preventively using one dry pad per day.

The routine removals of Foley catheter after S-RARP and T-RARP were done at postoperative 2 weeks and 1 week, respectively. As described in **Table 3**, the T-RARP group was related to a significantly higher proportion of patients achieving UC at the removal of catheter (90.0% vs. 30.0%, *p* < 0.001) and postoperative 3 months (100% vs. 58.3%, *p* < 0.001) than the S-RARP group. Among those with pT3a diseases, one and no case failed to return to UC at last follow-up in the S-RARP and T-RARP groups, respectively. Five and two patients were gradually exempt from the symptomatic complaint of nocturia by virtue of solifenacin succinate in the S-RARP and T-RARP groups, respectively. No drug interventions were applied on other patients complaining of urinary incontinence. However, the statistical differences in the rates of UC between the T-RARP and S-RARP groups turned to be insignificant at last follow-up (100% vs. 93.3%, *p* = 0.119). The cumulative incidence of postoperative UC recovery did significantly differ among patients following T-RARP and S-RARP (*p* = 0.001) (**Figure 2**).

**Figure 3:**
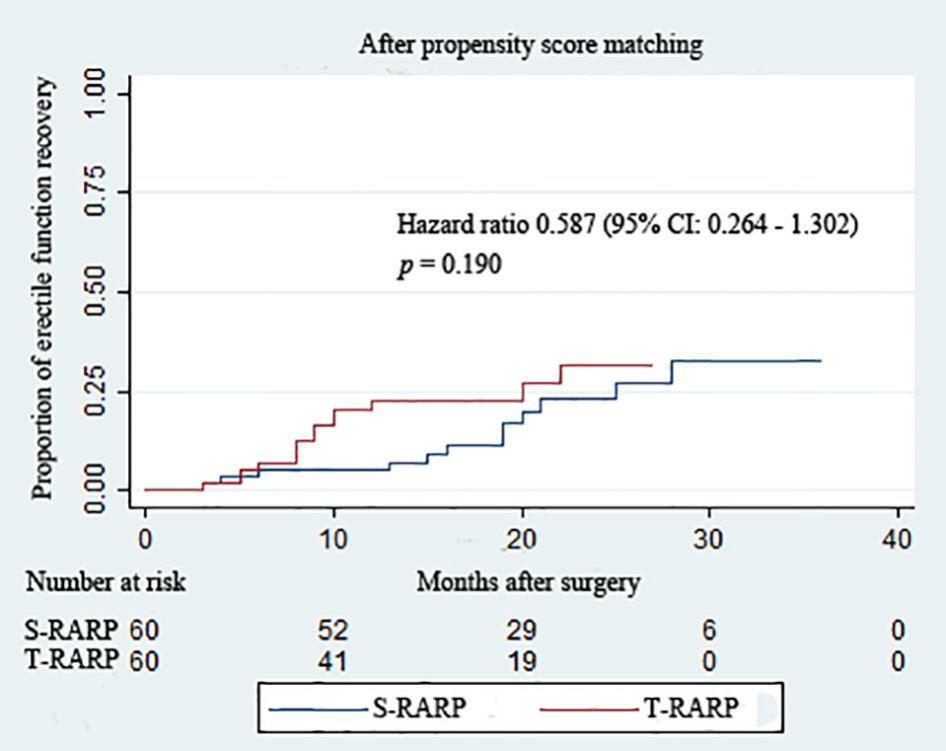
Kaplan–Meier curves showing the proportion of postoperative erectile function recovery according to surgical type during the follow-up intervals. Erectile function recovery was defined as a IIEF score ≥ 22.

Within the matched cohort, the median IIEF-5 score at postoperative 3 months and last follow-up didn’t differ significantly between the S-RARP and T-RARP groups (*p* = 0.322 and *p* = 0.471, respectively) (**Table 3**), and **Figure 3** depicts the similar estimated probability of EF recovery after T-RARP and S-RARP (*p* = 0.190), revealing the similar EF preservations of the two surgical procedures. Among those with pT3a diseases, 3 and 1 cases failed to recover to EF at last follow-up in the S-RARP and T-RARP groups, respectively.

## Discussion

Although there is a paucity of high-level evidences supporting the rapid diffusion of RARP, currently it has been the most extensively applied approach for prostatectomy (2, 3). Awareness has increased with respect to the slowly evolving nature of most localized prostate tumors and the significance of weighing treatment benefits and harms to avoid RP-related complications, especially the sexual and urinary dysfunctions which substantially decrease quality of life (14, 15). Multiple pathophysiologic mechanisms underlie the development of post-prostatectomy incontinence (PPI) (16, 17). In addition to the biological/preoperative elements consisting of patient age at time of surgery, pre-existing lower urinary tract symptoms, high BMI, and functional bladder changes, damages to anatomic support and pelvic innervation appear to be essential parameters in the occurrence of PPI (16). On the strength of the knowledge mentioned above, several innovative techniques, such as preservations of bladder neck (18) and neurovascular bundles (19), anterior reconstruction (20), and bladder neck plication (21), have been consequently adopted to maintain the intactness or to reply the functionality of periprostatic anatomic structures, thus accelerating UC recovery after prostatectomy. Unlike UC, EF anatomically and physiologically connects with the periprostatic neurovascular bundles in a clear manner (22). Nerve-sparing RP has been considered as the standard approach in all patients with normal EF and localized PCa to promote EF recovery (2, 22).

Although several published studies have demonstrated the advantage of the posterior approach in promoting early UC recovery over the standard approach (17, 23, 24), convincing evidences in oncologic control and long-term protection of urinary function after posterior RARP remain deficient. More serious problems coupled with the posterior method are that this approach is conducted under a smaller operative space and less and unfamiliar landmarks are provided when dissecting the lateral pedicles, thus making it impossible to view the position of the ureteral orifices after bladder neck division and challenging surgeons with an inverted relationship between bladder and prostate during dissection and reconstruction (24, 25). This predicament may be much more challenging when treating patients bearing large prostates or locally advanced disease with the posterior RARP, consequently resulting in an extremely extended learning curve (26). The biggest drawback of this access route is the underlying trend towards higher PSM rates when compared with the standard approach (5, 23, 24, 26, 27).

In this retrospective case series, we evaluated the oncological and functional outcomes of another innovative Retzius-sparing approach to RARP, namely T-RARP, which was firstly applied on patients afflicted with localized PCa by our team (10), in comparison with that of S-RARP. Given the preoperative confounding factors and selection bias, we applied the PM method to consolidate the comparability between the two groups. Within the matched setting, the results revealed that the enhanced postoperative UC rate and similar EF preservation were obtained after T-RARP without oncologic control being compromised when compared with S-RARP.

With respect to the similar perioperative outcomes containing EBL and rates of open conversion and transfusion acquired after the two techniques, the comparability might attributable to the similarity of the two surgical procedures, since the transvesical approach was carried out similarly to the standard method after the bladder neck excision. The familiarity to periprostatic anatomy under the three-dimensional magnified vision of robots could also contribute to these equivalent results in highly experienced hands. Benefiting from the high dexterity and clear visualization of robots, hemostasis could be timely and accurately performed during the surgeries, consequently helping to obtain comparable EBL and transfusion rate. The only two statistically significant differences in perioperative endpoints were found for OT and hospital stay length. However, the difference in OT of about 20 minutes has questionable clinical significance and may be explained by the surgeon’s learning curve, which was greatly reduced in experiences hands. Given the avoidance of pelvic drainage placement and the faster removal of catheter after T-RARP in a routine manner, the significant difference in hospital stay length could be well acceptable.

Within our matched cohort, the higher rate of tumors in pT3a stage and larger mean prostate volume, which usually related to more extended dissection, may help in interpreting the higher but insignificantly different proportion (15.0%) of postoperative complications in S-RARP group than that (8.3%) in the T-RARP group. Given the similarity in preserving the retropubic structures between T-RARP and posterior RARP, urinary catheter in the T-RARP group in our study was regularly removed on 7 days after surgery, which was similar to that following posterior RARP (28). The routine placement of pelvic drainage and the longer routine duration of indwelling urinary catheter in the S-RARP group could also translate into a higher risk of postoperative infection (29). In our analysis, the more frequent (3.3%) appearance of > Grade II complication (symptomatic lymphocele) after surgery could greatly blame on the more performances of ePLND in the S-RARP group, while the published probability of > Grade II postoperative complication after S-RARP ranged from 0% to 6.6% (5, 30, 31). The similarity in the tendency towards major postoperative complications demonstrated the safety of the two surgical procedures in managing localized PCa.

Our results declared the superiority of the transvesical approach (90%) over the standard approach (30%) concerning postoperative early return to UC. All key technical points of the transvesical approach were completed in the intentionally incised bladder without disrupting the integrity of Retzius space and recto-vesical pouch, thus providing a strong rationale for obtaining promising UC recovery. All UC-related structures in the Retzius space were spared to provide a strong supportive mechanism and stabilize the urethra (30, 32), such as the endopelvic fascia, puboprostatic ligaments and detrusor apron. After applying PM method, all biological/preoperative elements which may affect UC recovery were comparable between the T-RARP and S-RARP groups. Aside from these similar preoperative factors, surgeon’s experience was also perceived as a vital element in affecting prostatectomy outcomes (23). The extensive experience of the two surgeons involved in our study and the potential advantage of the transvesical method in generally high acquaintance among urologists could also partly ensure the superior outcomes in the T-RARP group. All mechanisms mentioned above could account for the faster return to UC after T-RARP than that after A-RARP. Given the reported rates of UC varying from 78% to 97.5% within postoperative 1 month after the posterior RARP (5, 33), our data exhibited the efficiency of T-RARP in strengthening early UC recovery. Interestingly, the significant difference in UC rates between the two groups was annihilated along with the gradual recovery to UC in the S-RARP group at the last follow-up, which could attribute to the gradually generated fibrosis surrounding the urethra as a supportive layer and reposition of the peritoneum and related structures which were damaged during S-RARP (30, 34). The proportion (30%) of UC on the removal of catheter in the T-RARP group of our series was consistent with that (28.7%) following RARP with posterior reconstruction in a prospective analysis (35) involving 803 patients with PCa, while the early UC rate obtained after RARP with posterior reconstruction ranged from 19.0% to 74.2% in published literatures (36, 37). The similarity between the transvesical and standard techniques in preserving the neurovascular structures during the initial proximity to the prostate and the insignificant difference in preoperative EF between the two groups could be greatly responsible for the comparable outcomes regarding EF.

Oncological control is of paramount significance when applying surgical innovation to manage solid cancer. Our study found a tendency towards a higher probability of PSM following T-RARP (15.0%) vs. S-RARP (11.7%) after matching, which may be attributable to the surgeon’s learning curve to T-RARP, a strongly contributing factor to PSM (38). Intriguingly, the statistical difference in the PSM rate between the T-RARP and S-RARP groups was insignificant, which may be explained by the fact that the procedure of T-RARP after bladder neck excision was carried out similarly to S-RARP. Given that the more extensive the cancer, the higher the risk of positive margins (38), the PSM rate obtained following the posterior RARP had reached up to 50% in pT3 diseases in the series reported by Abdel et al. (39). The relatively low rate of PSM in our T-RARP group could be partly interpreted with the extreme scarcity of pT3 prostate tumors included in our analysis, while the reported PSM rates after the posterior approach were 14-27% with more pT3 prostate tumors enrolled (5, 7, 39, 40). A larger working space provided by the suspension stitches during T-RARP was also conducive to achieve negative surgical margins when compared with the posterior approach to RARP. Although PSM was related to an enhanced hazard of biochemical relapse, the detrimental impact of PSMs on more robust clinical end points was fairly marginal relative to the impacts of Gleason score, pathologic stage, and preoperative PSA (5, 7, 38). In an analysis involving 5290 patients with PCa, Abdollah et al. (41) also noticed that PSMs, by themselves, were not independently related to enhanced hazards of clinical relapses in patients with localized diseases or Gleason score ≤ 7, which extremely represented the majority of our study population. Indeed, the BCR-free survival obtained after T-RARP tended to be similar to that after S-RARP, reflecting the comparable oncologic control of the two procedures.

Several notable limitations should be recognized when interpreting our findings. Structural deficiencies in gathering data were ineluctable in a retrospective manner in this analysis. The study population, although well-balanced between the two groups, is relatively small. We could not further estimate the long-term oncological survival and long-term functional outcomes over the relatively limited study duration. Certain complications may be underrated, especially ≤ Grade II complications, regardless of the elaborative investigation of medical records and telephone interview.

In spite of these limitations, our study represents a natural process during the development of a newly applied surgical technique. Our study is the first one designed to assess the perioperative, functional, and oncological outcomes acquired following T-RARP and S-RARP for localized PCa up to now, and our conclusions are drew and strengthened on the basis of the comparability of all perioperative elements between the two arms and rigorous methodology.

## Conclusions

T-RARP by experienced hands was feasible for selected patients with clinically localized prostate cancer, yielding significantly improved early return to UC and similar erectile functional preservation without compromising oncological control when compared with the standard approach. Our present conclusions need to be confirmed further on the basis of prospectively randomized trials with large sample sizes and sufficiently long follow-ups.

## Data Availability Statement

The raw data supporting the conclusions of this article will be made available by the authors, without undue reservation.

## Author Contributions

Conception and design: BF, GW, and XZ. Acquisition of data: WD, CZ, and HJ. Analysis and interpretation of data: XL and LC. Statistical analysis: WD and WL. Manuscript writing: WD. Manuscript editing: BF, GW, and XZ. All authors contributed to the article and approved the submitted version.

## Funding

This study was supported by the Natural Science Foundation of China (Grant No. 81760457).

## Conflict of Interest

The authors declare that the research was conducted in the absence of any commercial or financial relationships that could be construed as a potential conflict of interest.
